# Decision tree for accurate infection timing in individuals newly diagnosed with HIV-1 infection

**DOI:** 10.1186/s12879-017-2850-6

**Published:** 2017-11-29

**Authors:** Chris Verhofstede, Katrien Fransen, Annelies Van Den Heuvel, Kristel Van Laethem, Jean Ruelle, Ellen Vancutsem, Karolien Stoffels, Sigi Van den Wijngaert, Marie-Luce Delforge, Dolores Vaira, Laura Hebberecht, Marlies Schauvliege, Virginie Mortier, Kenny Dauwe, Steven Callens

**Affiliations:** 1 Aids Reference Laboratory, Department of Clinical Chemistry, Microbiology and Immunology, Ghent University, Ghent, Belgium; 2 HIV/STD Reference Laboratory, Department of Clinical Sciences, Institute of Tropical Medicine, Antwerp, Belgium; 3 Department of Microbiology and Immunology, Rega Institute for Medical Research, KU Leuven – University of Leuven, Leuven, Belgium; 4 Aids Reference Laboratory, University Hospitals Leuven, Leuven, Belgium; 5 Aids Reference Laboratory, Medical Microbiology unit, Université Catholique de Louvain, Brussels, Belgium; 6 Aids Reference Laboratory, Vrije Universiteit Brussel VUB, Brussels, Belgium; 7 Aids Reference Laboratory, Centre Hospitalier Universitaire St. Pierre, Brussels, Belgium; 8 Aids Reference Laboratory, Université Libre de Bruxelles, Brussels, Belgium; 9 Aids Reference Laboratory, Centre Hospitalier Universitaire de Liège, Liège, Belgium; 10 Aids Reference Center and Department of Internal Medicine, Ghent University Hospital, Ghent, Belgium

**Keywords:** HIV, Infection timing, Incidence measurement

## Abstract

**Background:**

There is today no gold standard method to accurately define the time passed since infection at HIV diagnosis. Infection timing and incidence measurement is however essential to better monitor the dynamics of local epidemics and the effect of prevention initiatives.

**Methods:**

Three methods for infection timing were evaluated using 237 serial samples from documented seroconversions and 566 cross sectional samples from newly diagnosed patients: identification of antibodies against the HIV p31 protein in INNO-LIA, SediaTM BED CEIA and SediaTM LAg-Avidity EIA. A multi-assay decision tree for infection timing was developed.

**Results:**

Clear differences in recency window between BED CEIA, LAg-Avidity EIA and p31 antibody presence were observed with a switch from recent to long term infection a median of 169.5, 108.0 and 64.5 days after collection of the pre-seroconversion sample respectively. BED showed high reliability for identification of long term infections while LAg-Avidity is highly accurate for identification of recent infections. Using BED as initial assay to identify the long term infections and LAg-Avidity as a confirmatory assay for those classified as recent infection by BED, explores the strengths of both while reduces the workload. The short recency window of p31 antibodies allows to discriminate very early from early infections based on this marker. BED recent infection results not confirmed by LAg-Avidity are considered to reflect a period more distant from the infection time. False recency predictions in this group can be minimized by elimination of patients with a CD4 count of less than 100 cells/mm3 or without no p31 antibodies. For 566 cross sectional sample the outcome of the decision tree confirmed the infection timing based on the results of all 3 markers but reduced the overall cost from 13.2 USD to 5.2 USD per sample.

**Conclusions:**

A step-wise multi assay decision tree allows accurate timing of the HIV infection at diagnosis at affordable effort and cost and can be an important new tool in studies analyzing the dynamics of local epidemics or the effects of prevention strategies.

## Background

Measurement of HIV incidence is an essential instrument for reliable monitoring of the effect of preventive interventions like generalized treatment, early treatment, pre-exposure prophylaxis or vaccines. HIV surveillance in Western-European countries is mostly based on case reporting of new cases but defining the infection prevalence is less well suited for tracking trends in HIV transmission as it is prone to biases resulting from shifts in testing rates or changes in populations at risk. UNAIDS provides guidelines for incidence measurement [1] and many countries have programs for nationwide incidence measurement. Despite all these efforts, a gold standard method for the timing of an HIV infection at diagnosis is still missing. The only reliable way to classify an infection as recent is the demonstration of a seroconversion, but the seroconversion period is short and HIV diagnosis often delayed. Different approaches for HIV incidence measurement have been proposed, using either information from routine diagnostic assays [2], specialized assays [3–5] or mathematical models [6]. While all these models may be useful for incidence measurement in large populations, their value for the estimation of time of infection in individual patients is limited. Also, the multitude of approaches used for incidence measurement today hampers comparison of incidence rates between studies and countries.

Several assays have been developed that rely on characteristics of the humoral immune response after infection to estimate recency of infection, such as the increase in anti-HIV IgG antibody concentration relative to total IgG concentration and the increase in anti-HIV antibody affinity. Ideally, these assays should have a false recency rate of less than 2% [7]. The struggle to achieve this goal hinders routine implementation of these assays [7–11] and oblige the exclusion of patients known to have higher risk for false recent classifications, to improve accuracy. Alternatively, incidence measures can be optimized by the use of algorithms that rely on multiple parameters and as such correct for the inaccuracies of individual assays.

Systematic incidence analysis also has logistic and financial implications especially when multiple assays are involved. Serial testing approaches or decision trees maintain accuracy but control costs by reducing the number of samples for extensive testing [12]. They are an interesting solution but need to be carefully designed to avoid loss of information. The aim of this study was to explore different testing schemes for HIV incidence measurement and surveillance in Belgium. This country has a relatively high HIV prevalence, with a stable 90 to 100 new diagnoses per million inhabitants per year since 2003. Men having sex with men account for 46% of all new diagnoses, heterosexual transmission for 50%. Sub-Saharan African migrants represent 45% of the new diagnoses resulting from heterosexual contacts [13]. The heterogeneity in origin of infected individuals is reflected in a multitude of HIV subtypes and recombinants being represented. Confirmation of reactive HIV screening is centralized in 7 reference laboratories who report new diagnoses encoded to the Institute of Public Health. The reference laboratories are also in charge of the laboratory monitoring of HIV infected patients and as such have access to viral load and CD4 count data for all patients entering care.

The following markers of infection time were evaluated: absence of antibodies against the HIV-1 p31 antigen as visualized on the immunoblot confirmation test (INNO-LIA HIV I/II Score, Fujirebio, Ghent, Belgium), low HIV IgG concentration as defined by Sedia™ BED HIV-1 Incidence Enzyme Immuno Assay (BED-CEIA; Sedia Biosciences Corporation, Portland, Oregon, USA) and low HIV antibody avidity as defined by Sedia™ HIV-1 Limiting Antigen Avidity Enzyme Immuno Assay (LAg-Avidity EIA; Sedia Biosciences Corporation). Strengths and weaknesses of these markers alone and in combination were defined on serial samples from seroconverters, on samples from patients with particular conditions and on cross-sectional samples from the target population. Based on the obtained results a decision tree for future infection timing was developed.

## Methods

### Study population

Table 1 presents an overview of patient characteristics for the different sample series.

**Table 1:** Characteristics of patients in the different sample series

Sample series		Patients (n)	Samples (n)	Samples/ patient	Male (%)	Age at diagnosis mean (IQR)	Subtype B (%)	CD4 count cells/mm^3^ mean (IQR)	Log viral load mean (IQR)
Longitudinal		42	237	4 to 15	85.7	38 (33 - 44)	70.7	612 (461 - 709)^a^	5.18 (4.58 - 5.75)^a^
Special populations	ART treated	12	12	1	50.0	45 (38 - 55)	NA	694 (429 - 998)	<1.30
	Elite controllers	11	11	1	27.3	49 (38 - 57)	NA	933 (769 - 1081)	<1.30
	Slow progressors	6	6	1	50.0	41 (36 - 50)	NA	599 (374 - 883)	2.45 (2.23 - 2.72)
	Advanced infection	19	19	1	73.7	39 (34 - 45)	58.8	42 (14 - 61)	5.40 (4.98 - 5.72)
Cross-sectional		566	566	1	78.3	39 (30 - 47)	53.0	443 (264 - 566)	4.71 (4.26 - 5.22)

*Abbreviations*: *ART* combination antiretroviral therapy, *IQR* interquartile range, *n* number, *NA* not available

^a^Using only the result of the first sample collected from each patient

The longitudinal sample series comprised 237 serial plasma samples from 42 patients with a documented seroconversion. The mean number of samples tested per patient was 7 (range 4 to 15) and the mean total follow-up period was 696 days (range 67 to 2364 days). The first sample collected from each patient (day 0) showed a reactive p24 antigen test but no detectable antibodies in the INNO-LIA HIV I/II Score.

The cross-sectional sample series consisted of single samples collected at diagnosis or a maximum of 6 weeks thereafter from 566 individuals diagnosed with HIV-1 in Belgium between 2012 and 2014. All patients were therapy naïve and had a detectable viral load (>20 c/ml) at the time of sample collection. The HIV subtype was available for 511 individuals, the majority (53.0%) were infected with a subtype B virus. The other subtypes and circulating recombinant forms (CRF) represented were CRF02_AG (*n* = 74), F (*n* = 63), A (*n* = 33), C (*n* = 26), CRF01_AE (*n* = 17), G (*n* = 11), other CRF (*n* = 9) and D (n = 7).

The sample series of patients with particular conditions known to be associated with a higher likelihood for false recency predictions contained single samples from 12 patients on antiretroviral therapy (ART) (treated for at least 6 months, viral load <20 copies/ml (c/ml)), from 11 elite controllers (infected for at least 4 years, therapy naïve, viral load <20 c/ml), from 6 slow progressors (infected for at least 4 years, therapy naïve, viral load between 20 and 1000 c/ml) and from 19 advanced infections (infected more than 5 years, viral load >30,000 c/ml, CD4 count <100 cells/mm^3^).

All samples were selected from the serum and plasma depository of the 7 Belgian Aids Reference Laboratories. The study was approved by the ethical committee of the participating institutions with Ghent University Hospital as the leading center (study number 2014/0717).

### INNO-LIA and p31 antibodies

The INNO-LIA HIV I/II Score assay (Fujirebio) was performed according to the manufacturers’ instructions. Using the band intensity of the three internal standards, antibody reaction to the individual HIV antigens was scored as -, ±, 1+, 2+ or 3+ either visually or by the automated LIRAS reading system (Fujirebio). Antibodies against the integrase protein p31 were considered absent when a score of **–** or ± was given and present when the score was 1+ or more. For the longitudinal samples, INNO-LIA was performed on serial samples until presence of p31 antibodies was documented. For the samples from selected populations an INNO-LIA was performed in the context of this study, for the cross-sectional samples from newly diagnosed patients the results of the diagnostic assay were used.

### LAg-avidity EIA and BED CEIA

Sedia™ BED CEIA (Sedia Biosciences Corporation, Portland, Oregon, USA) and Sedia™ HIV-1 LAg-Avidity EIA (Sedia Biosciences Corporation), were performed according to the manufacturers’ instructions. Results are normalized using an internal calibrator and reported as normalized optical densities (OD-n). Attribution of long term infections was based on a single measurement and OD-*n* > 1.2 for BED CEIA or >2.0 for LAg-Avidity EIA. For samples with OD-n ≤ these respective cut-offs, retesting in triplicate was performed as recommended and discrimination between recent and long term infection was based on median OD-*n* > 0.8 for BED CEIA and >1.5 for LAg-Avidity EIA.

### Subtype assignment

Sequences of the HIV-1 *protease* and *reverse transcriptase* genes gathered for the purpose of baseline resistance testing were used for subtype assignment using Rega v3, http://dbpartners.stanford.edu:8080/RegaSubtyping/stanford-hiv/typingtool/ (Rega Institute for Medical Research, Leuven, Belgium) [14] and Comet, http://comet.retrovirology.lu/ (Laboratory of Retrovirology, Luxembourg Institute of Health, Luxembourg) [15]. The subtype was only allocated in case of concordant outcome of both tools and considered as undefined (UD) in case of discordancy. The sequences were assigned Genbank accession numbers MF754381 to MF754919.

### Statistical analysis

Rosner’s Extreme Studentized Deviate (ESD) test with a significance level set at 0.05 was used to identify outliers (https://www.medcalc.org/manual/outliers.php; last accessed on November, 29th 2017). Fisher’s exact test was used to compare the representation of patients with low CD4 count amongst the congruent and incongruent predictions.

## Results

### Serial samples from seroconverters

**Fig. 1:**
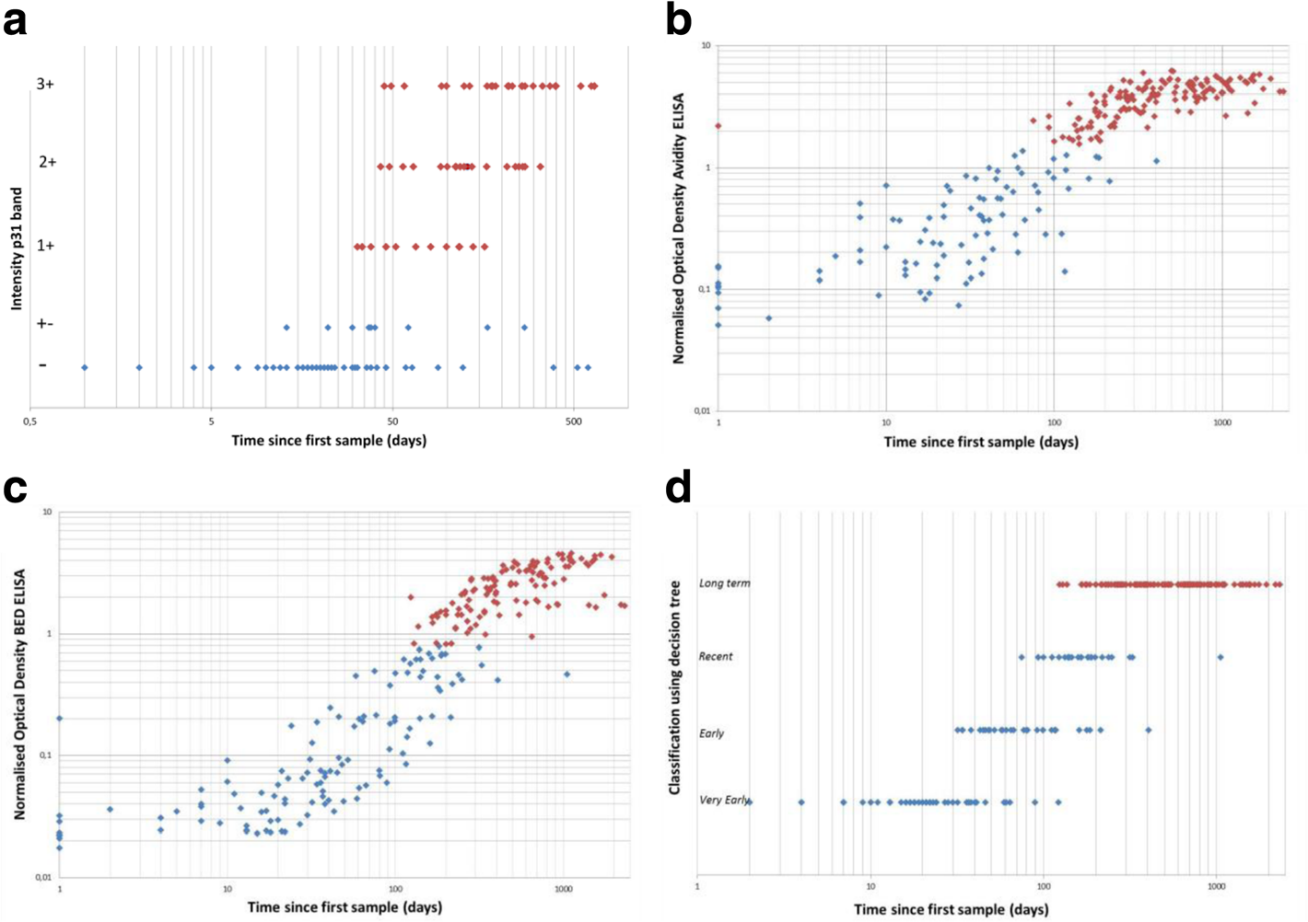
Results of p31 antibody presence (**a**), LAg-Avidity EIA (**b**), BED CEIA (**c**) and the decision tree (**c**) in seroconverters. Scatterplots represent the p31 intensity on INNO-LIA strips (**a**), the normalized optical density for the LAg-Avidity EIA (**b**), the normalized optical density for BED CEIA (**c**) and the decision tree classification (**d**) for 237 samples from 42 seroconverters. The time of the first sample, collected pre-seroconversion, is considered as day 0. Blue diamonds represent classification as recent infection; red diamonds represent classification as long term infection

For the longitudinal study, the day of collection of the first (pre-seroconversion) sample was considered as day 0. Antibodies against the p31 antigen were absent in 28.7% of the samples and were first detected in a sample collected at day 32. The median time point between the last sample without p31 antibodies and the first sample with p31 antibodies was day 64.5 (IQR 33.5 – 89.0) (Fig. 1a). With the LAg-Avidity EIA, 38.8% of the serial samples were classified as recent and 61.2% as long term (Fig. 1b). The samples from recent infections were collected between day 0 and 406. The median time point between recent and long term classifications was day 108.0 (IQR 89.5–157.5) (Fig. 1b). With the BED CEIA, 50.0% of the samples were classified as recent and 50.0% as long term (one sample was not analyzed with BED CEIA). The recent infections were collected between day 0 and 1053. The median time point between recent and long term classification was day 169.5 (IQR 108.0 – 234.5) (Fig. 1c).

**Fig. 2:**
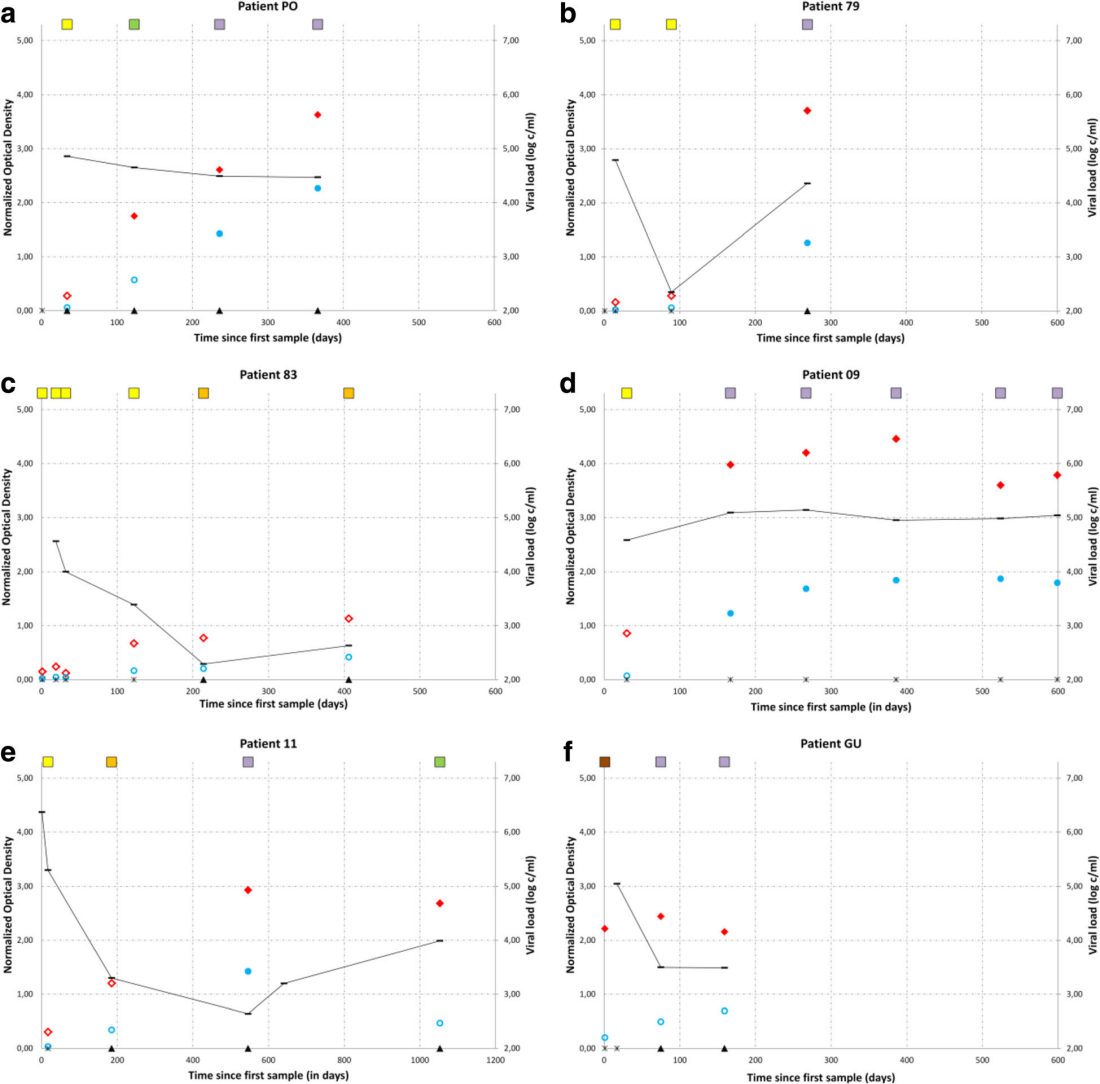
Assay results in function of time since collection of the first sample for 6 demonstrative patients. **a** represents the evolution of the markers in a representative patient (PO) and the **b** to **f** represent the evolution in all patients with at least one outlier result. Black stars: absence of p31 antibodies; black triangles: presence of p31 antibodies. Blue circles: BED CEIA results, open circles: recent infection, solid circles: long term infection. Red diamonds: LAg-Avidity EIA results; open diamonds: recent infection, solid diamonds: long term infection. Black line: evolution of the viral load. Squares on top of the graphs represent the results obtained when following the decision tree, yellow: very early infection, orange: early infection, green: recent infection, purple: long term infection and brown: advanced infection

Figure 2a shows the evolution of assay results over time in a representative patient (patient PO). Rosner’s ESD test identified 7 outliers for p31 antibodies, 3 for LAg-Avidity EIA and 2 for BED CEIA. Absence of p31 antibodies was noticed in samples collected at day 89, 122, 167, 267, 386, 524 and 599 reflecting delayed p31 antibody production in the 3 patients from whom they were drawn: patients 79 (Fig. 2b), patient 83 (Fig. 2c) and patient 09 (Fig. 2d). The LAg-Avidity EIA falsely classified a sample collected at day 0 as long term infection: patient GU Fig. 2e) and 2 samples collected from patient 83 at day 214 and 406 as recent infection (Fig. 2c). BED CEIA falsely classified a sample from day 406 (patient 83, Fig. 2c) and a sample from day 1053 (patient 11, Fig. 2f) as recent infection.

The 5 patients with outlier results were all infected with a subtype B virus. Three of the 4 patients with an extended recency window for either both EIAs and p31 antibodies (patient 83, Fig. 2d), BED CEIA alone (patient 11, Fig. 2e) or p31 antibodies alone (patient 79, Fig. 2f) also showed a pronounced viral control after acute infection in the absence of cART. The overall sensitivity and specificity of the two EIA assays when applying different cut-offs for duration of infection is summarized in Table 2.

**Table 2:** Sensitivity and specificity of LAg-Avidity, BED and the decision tree for longitudinal seroconverter samples

							Decision tree			
Duration of infection (days)	Recent infection (n)	Long term infection (n)	LAg-Avidity EIA		BED CEIA		Classification 1		Classification 2	
			Sensitivity	Specificity	Sensitivity	Specificity	Sensitivity	Specificity	Sensitivity	Specificity
110	87	150	0.94	0.93	1.00	0.79	0.95	0.93	1.00	0.79
120	92	145	0.93	0.96	1.00	0.81	0.95	0.96	1.00	0.81
130	96	141	0.91	0.96	0.98	0.82	0.92	0.96	0.98	0.82
140	99	138	0.88	0.96	0.97	0.83	0.89	0.96	0.97	0.83
150	103	134	0.84	0.96	0.97	0.86	0.85	0.96	0.97	0.86
160	104	133	0.84	0.96	0.97	0.86	0.85	0.96	0.97	0.86
170	109	128	0.81	0.97	0.95	0.88	0.82	0.97	0.95	0.88
180	113	124	0.79	0.98	0.93	0.89	0.80	0.98	0.93	0.89

Different recency windows were applied by changing the cut-off for duration of infection. The duration of infection is the periods (in days) after collection of the first pre-seroconversion sample. Recent infection: samples taken before the cut-off set for duration of infection, long term infection: samples taken at or after the cut-off set for duration of infection. For the decision tree, either all ‘very early’ and ‘early’ results were considered as recent (classification 1) or all ‘very early’, ‘early’ and ‘recent’ results were considered as recent (classification 2)

*Abbreviations*: *n* number, *EIA* Enzyme Immuno Assay, *CEIA* Capture Enzyme Immuno Assay

Of note, 2 patients who initiated cART during the acute phase of infection showed an extended recency windows for all 3 markers (analyzed outside of study, results not shown).

### Elite controllers, slow progressors, ART treated and late stage disease patients

High rates of false recent predictions were observed in patients on ART and in elite controllers (Table 3), with BED CEIA suffering the most from this type of misclassification, followed by LAg-Avidity EIA and p31 antibodies. Advanced disease and low CD4 count were also associated with a high number of false recent predictions in BED CEIA and p31 antibody assay but not in LAg-Avidity EIA (Table 3). No false recent classifications were noticed for the slow progressors.

**Table 3:** False Recency predictions in special populations

	n	INNO-LIA p31 (%)	LAg-Avidity EIA (%)	BED CEIA (%)	Decision tree (%)
ART treated	12	16.7	41.7	100.0	100.0
Elite controllers	11	27.3	36.4	36.4	36.4
Slow progressors	6	0	0	0	0
Advanced infections	19	36.8	0	21.1	0

*Abbreviations*: *ART* Antiretroviral Therapy, *EIA* Enzyme Immuno Assay, *CEIA* Capture Enzyme Immuno Assay

### Newly diagnosed individuals

An overview of the results for the 566 cross-sectional samples from newly diagnosed patients is shown in Table 4. Concordant recent infection classifications for 3 markers were obtained for 105 (18.9%) patients and concordant long term infection classifications for 313 (55.3%), resulting in an overall concordance of 73.9%. When comparing the results of the two EIAs, the concordance was 85.3%, with 28.4% of the samples classified as recent infections and 56.9% as long term infections. Discordances between the EIA results were observed for 83 patients (14.7%), 82 classified as recent by BED and long term by LAg-Avidity and 1 classified as recent by LAg-Avidity and long term by BED.

**Table 4:** Result of infection timing for cross-sectional samples from 566 newly diagnosed individuals

INNO-LIA	p31	LAg-Avidity EIA	BED CEIA			Decision tree					
				n		Seroconversion	Very early	Early	Recent	Long term	Advanced
Negative/ID	Recent	Recent	Recent	24	Congruent	24					
Positive	Recent	Recent	Recent	81			81				
Positive	Long-term	Recent	Recent	56				56			
Positive	Long-term	Long-term	Recent	74					71		3
Positive	Long-term	Long-term	Long-term	313						313	
Positive	Recent	Long-term	Recent	8	Incongruent						8
Positive	Long-term	Recent	Long-term	1						1	
Positive	Recent	Long-term	Long-term	9						9	
				566							

Results that are in-line with the expectations when considering the differences in recency window are labeled as ‘congruent’ and results that are not in-line with these expectations are labeled as ‘incongruent’

*Abbreviations*: *ID* indeterminate, *EIA* Enzyme Immuno Assay, *CEIA* Capture Enzyme Immuno Assay, *INNO-LIA* Innogenetics Line Immuno Assay

For subsequent analysis, results that followed the expectations considering the differences in recency window between the assays were called congruent and results that could not be explained by the recency window were called incongruent (Table 4). The total number of incongruent predictions was low (*n* = 18, 3.2%) with the highest number resulting from false recent predictions, 17 for p31 antibodies, 8 for BED CEIA and 1 for LAg-Avidity EIA.

To further identify potential reasons for incongruences, viral load and subtype distribution were compared between the classification groups. The results showed that incongruences were observed significantly more frequently in patients with a low CD4 count (<200 cells/mm^3^; *p* < 0.01) but influence of viral load or subtype was not significant. Of note, 7 of the 9 patients classified as long term infections by both EIAs but in whom p31 antibodies were absent were subtype B infections.

### Adaptation of cut-offs for retesting in triplicate

The standard protocol for BED CEIA and LAg-Avidity EIA recommend triplicate retesting of all samples with a OD-n of ≤1.2 and ≤2.0 respectively. The added value of this confirmatory test was investigated by analyzing the number of samples with a different conclusion after triplicate testing than when only the initial singleton test result would have been taken into account. This was the case for 4 samples for BED CEIA and 13 samples for LAg-Avidity EIA. These samples had an initial singleton OD-n between 0.706 and 0.881 for BED CEIA and between 1.160 and 1.553 for LAg-Avidity EIA. For the decision tree we therefore felt confident to narrow the range of OD-n requiring confirmatory retesting to ≤1.000 and >0.700 for BED and ≤1.600 and >1.100 for LAg-Avidity.

### Decision tree

**Fig. 3:**
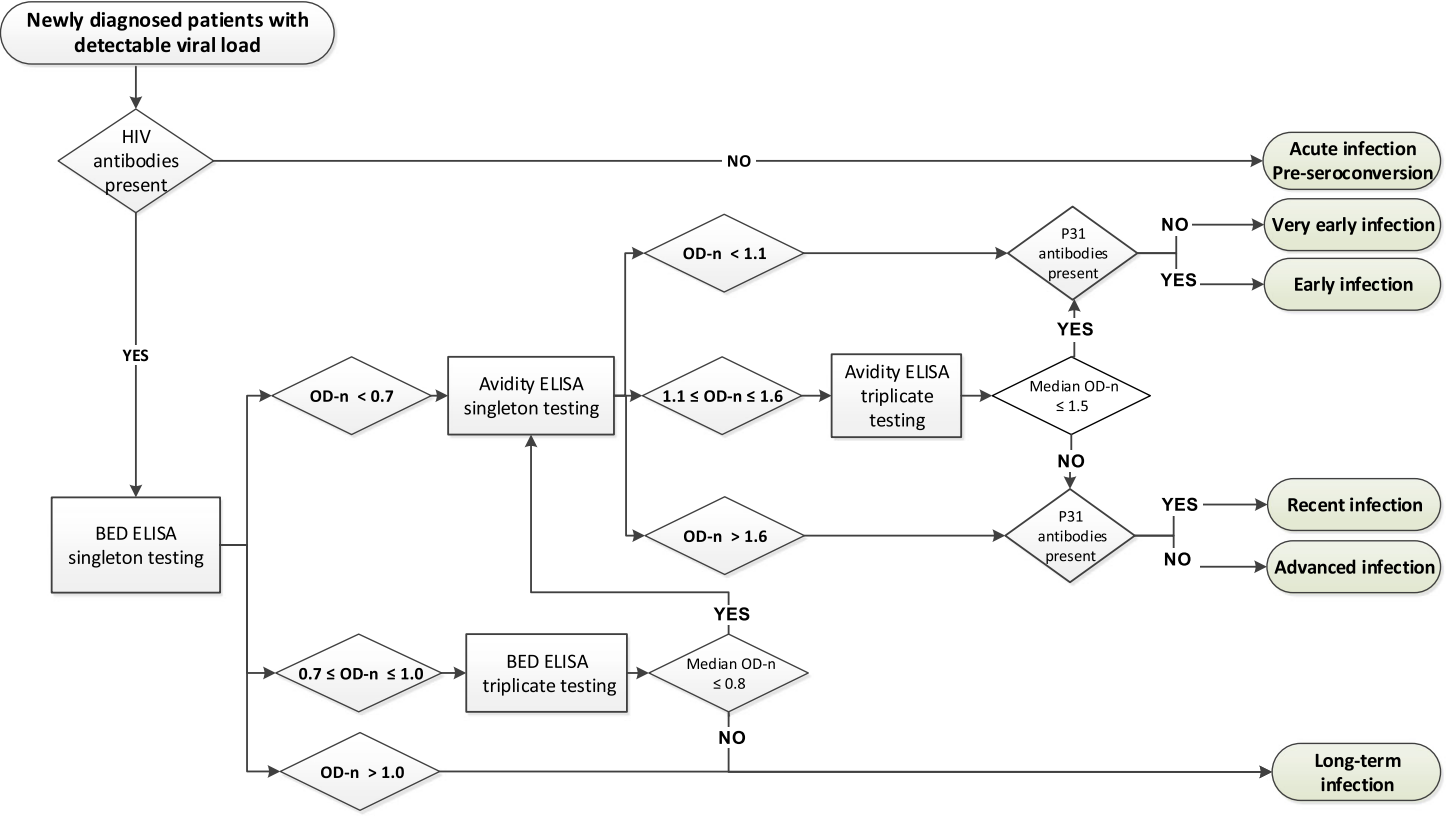
Decision tree for infection timing

Based on the assembled information a multi-assay decision tree for infection timing at diagnosis in therapy naïve patients with a detectable viral load was designed (Fig. 3). The initial step is identification of pre-seroconversion samples (negative or indeterminate INNO-LIA) and the immediate classification of these patients as acute infections. Next all remaining samples are tested with the BED CEIA using the adapted cut-offs for triplicate retesting to identify long term infections. Samples predicted by BED CEIA as recently infected are then tested in LAg-Avidity EIA. The LAg-Avidity results are interpreted together with information on presence or absence of p31 antibodies to identify patients with a ‘very early’ infection (both EIAs indicate recent infection, p31 antibodies still absent) and patients with an ‘early’ infection (both EIAs indicate recent infection, p31 antibodies present). Samples with a BED recent-LAg-Avidity long term prediction, are considered to be collected in a slightly more advanced infection stage called ‘recent’. The latter classification is most prone to errors. To reduce the number of false recent infection classifications, samples in this categories without p31 antibodies or with a CD4 count of <100 cells/mm^3^ are reclassified as presumed advanced infections. Considering the calculated recency windows for both EIA and p31 antibodies it is estimated that the very early classification represent infections of less than 3 months, the early classification infections of less than 4 months and the recent classification infections of less than 6 months. For the serial samples from seroconverters, the decision tree was able to time the infection with a sensitivity and specificity identical to or higher than the sensitivity and specificity obtained for the individual assays. The results obtained after simulation of the decision tree for the cross-sectional samples are shown in Table 4. For these 566 cross-sectional samples, application of the decision tree would have reduced the number of single tests with 29.8% and the number of triplicate tests with 84.2% resulting in an estimated cost reduction per sample from 13.2 USD to 5.2 USD, without any loss in information.

## Discussion

Acute HIV-1 infection is characterized by the presence of viral antigens or viral RNA in the absence of specific antibodies and can easily be recognized based on the results of routinely performed diagnostic assays [16]. The sequential emergence of reactivity in different diagnostic tests has been used as a tool for the staging of primary infection by Fiebig et al. [17]. The acute pre-seroconversion stage however is short, considered to last between 12 and 99 days [18], and most infections are diagnosed later. Considerable effort has been devoted to the validation of methods or approaches that allow reliable estimation of the time of infection at diagnosis post-seroconversion. The literature on this subject is extensive but lacks consistency, with regard to the methods applied, the assays used and the patient population tested. Despite the many efforts, a generally accepted gold standard method for infection timing is still missing. Any initiative for HIV incidence measurement therefore requires appropriate validation of potential approaches on samples collected from the intended target population.

The aim of the present study was to develop a method for infection timing in patients newly diagnosed with HIV-1 infection in Belgium. The objective was to find an approach that permits incidence measurement but also enables reliable infection timing in individual patients. The latter must facilitate a more profound characterization of patients entering care, a better identification of the reasons for delayed diagnosis and improved insight in the period during which a patient is most vulnerable to transmit his virus to a second party. This information is important for the follow-up, adjustment and improvement of preventive efforts.

Three known markers of infection time were selected for validation; the HIV specific antibody concentration measured by BED CEIA, the HIV antibody affinity measured by LAg-Avidity EIA and the p31 antibody presence or absence defined from the readout of the INNO-LIA confirmatory assay. In analogy with the method of Fiebig et al. [17] we then used the differences in recency window between these assays to design a decision tree for serial testing that can distinguish between different stages of the early infection at low effort and costs. Serial samples from acutely infected individuals were tested in order to define the average time that an infection is classified as recent (the recency window) by the different markers. The sensitivity and specificity obtained for the LAg-Avidity and BED EIA approached those reported before [19]. Clear differences in de median duration of recent infection were observed between the markers, with calculated recency windows of 64.5 days (for p31 antibodies), 108.0 days (for LAg-Avidity EIA) and 169.5 days (for BED CEIA). For both EIAs, the calculated recency window approaches the cut-off provided by the manufacturer; 130 days for LAg-Avidity and 162 to 197 days for BED. The difference of about 30 days can be explained by the fact that we considered the day of collection of the first sample as day 0 and not the day of the infection. Others have reported comparable recency windows [20] but some observed a longer duration of recent infection [7, 21]. Individual differences between patients and the composition of the population used for validation may, to some extent, influence the mean recency window. E.g. we noticed longer recency windows in patients that are able to control the viral load to a lower set point early after infection. Incidence EIAs from different manufacturers may have slightly different recency windows, even if they are testing the same parameter and also the method used for calculation of the recency window may differ.

Since the recency window highly impacts calculated incidence rates, it is an essential factor to consider when deciding on the use of an assay for infection timing. The most ideal recency window for incidence calculation is one year but none of the markers evaluated reached that time limit. The BED CEIA has the longest recency window and thus seems the most suited but, as we and others observed, it is also the assay with the highest false recency rate [22–24]. In a recent publication, Kassanjee et al. suggest to optimize the recency windows of immunoassays by tuning of the thresholds [25]. This approach, that was also explored by Konikoff et al. [26] deserves further exploration.

To minimize false classifications, the BED CEIA manufacturer recommends exclusion of individuals on ART, elite suppressors and individuals with a CD4 count <200 cells/mm^3^. While our findings support the importance of excluding patients with an undetectable viral load, the added value of eliminating patients with a CD4 count <200 is debatable. Though this will eliminate part of the false recent predictions in advanced infection [27], it will fail to exclude them all. Besides, because the CD4 count may occasionally drop to below 200 cells/mm^3^ after acute infection [28, 29], exclusion of patients with a low CD4 count may impact the figures on recent infections. We therefore opted to only reclassify as advanced infections those patients with a CD4 count of less than 100 cells/mm^3^ that are considered as recently infected by BED CEIA only. False recent predictions of BED EIA in the advanced stage of infection can be explained by the reduction in HIV antibody concentration with progressing disease [30]. This decrease in antibody concentration may also lead to disappearance of p31 antibodies but seems to have little effect on the overall antibody affinity. Indeed, false recent predictions in patients with late stage disease are rare for the LAg-Avidity EIA [7, 19, 31]. In the decision tree, absence of p31 antibodies in patients identified as recently infected only by BED CEIA is considered indicative for the advanced stage of infection. We cannot exclude however that patients failing to mount a p31 antibody response or with a delayed p31 antibody production, as one of the patients in the longitudinal study, are falsely considered as having an advanced disease but because this is considered to be a rare phenomenon we believe that this potential error does not outweigh the overall gain in reliability of the infection timing.

The choice for BED CEIA in first line is justified by its high reliability for the identification of long term infections and its lower price (approximately 450 USD per kit versus 650 USD for LAg-Avidity EIA). The use of BED CEIA in first line also allows to profit from the longer recency window of this assay and provides a way to discriminate between ‘early’ and ‘recent’ infections. The classifications ‘very early’ or ‘early’ infection will always be based on a predicted recent infection by both EIAs and is therefore highly reliable. The classification ‘recent’ infection will be only based on a predicted recent infection by the BED CEIA, an assay with a higher false recent rate. Despite a correction for false receny predictions based on the CD4 count and the p31 antibody presence or absence this category may suffer from some erroneous classifications. For this study the information on the presence or absence of p31 antibodies is deduced from the INNO-LIA confirmatory assay. Whether the more recently developed immunochromatographic assay, Geenius**™** HIV ½ supplemental assay (Biorad, Marnes-la-Coquette, France) may serve this purpose needs to be examined.

This study was performed in a country with a heterogeneous HIV epidemic [13, 32]. There have been some reports about subtype related differences in the performance of incidence EIAs, more particularly a higher frequency of false recent predictions by BED CEIA in subtype D has been described [33, 34]. We found no indications for influence of the subtype on the infection timing but the contribution of subtype D was low. Of the 7 subtype D infections, 3 were predicted as long term, 1 as very early, 2 as early and 1 as recent. Unexpectedly, a higher contribution of subtype B infections was observed amongst the patients predicted as long term infected by both EIAs but missing p31 antibodies. The overrepresentation of subtype B in these patients with a presumed advanced disease was not statistically significant but further investigation is warranted.

Any incidence measurement that is based on specific features of the humoral immune response will be prone to errors because of the natural variability in antibody production and maturation. Quantification of the genetic variability of the virus has been suggested as an alternative marker of infection time [35] but the methods to define genetic variability are cumbersome and difficult to standardize and it can be expected that the extent of genetic evolution will also vary significantly between individuals.

Serial testing algorithms for HIV incidence measurement have been presented before [12, 36]. Using this approach we developed a decision tree that, with limited effort and cost, combines accuracy and precision to define HIV-1 infection time on single samples.

## Conclusion

Taken into account the strengths and weaknesses of different markers of infection time, a stepwise decision tree was set up that allows to classify, at diagnosis, an HIV infection as very early (infection of less than 3 months before), early (less than 4 months before) and recent (less than 6 months before). Being able to discriminate these early infection phases will be an important asset in studies aimed at analyzing the dynamics of local epidemics and the effects of prevention strategies.

## Abbreviations

ART: Antiretroviral therapy
BED: Antigen of HIV subtype B, E and D
CD4: Cluster of differentiation 4
CEIA: Capture enzyme immuno assay
EIA: Enzyme immuno assay
ESD: Extreme studentized deviate
HIV: Human immunodeficiency virus
INNO-LIA: Innogenetics line immuno assay
IQR: Inter quartile range
OD: Optical density
OD-n: Normalized optical density
P24: HIV protein 24 (capsid protein)
P31: HIV protein 31 (integrase)
UNAIDS: United Nations Programme on HIV/AIDS
USD: US dollar

## References

[1] UNAIDS/WHO Working Group on Global HIV/AIDS and STI Surveillance: When and how to use assays for recent infection to estimate HIV incidence at a population level. 2011. http://www.who.int/diagnostics_laboratory/hiv_incidence_may13_finalpdf. Accessed 8 Aug 2017.

[2] Schupbach J, Bisset LR, Gebhardt MD, Regenass S, Burgisser P, Gorgievski M, Klimkait T, Andreutti C, Martinetti G, Niederhauser C (2012). Diagnostic performance of line-immunoassay based algorithms for incident HIV-1 infection. BMC Infect Dis.

[3] Dobbs T, Kennedy S, Pau CP, McDougal JS, Parekh BS (2004). Performance characteristics of the immunoglobulin G-capture BED-enzyme immunoassay, an assay to detect recent human immunodeficiency virus type 1 seroconversion. J Clin Microbiol.

[4] Suligoi B, Galli C, Massi M, Di Sora F, Sciandra M, Pezzotti P, Recchia O, Montella F, Sinicco A, Rezza G (2002). Precision and accuracy of a procedure for detecting recent human immunodeficiency virus infections by calculating the antibody avidity index by an automated immunoassay-based method. J Clin Microbiol.

[5] Eshleman SH, Hughes JP, Laeyendecker O, Wang J, Brookmeyer R, Johnson-Lewis L, Mullis CE, Hackett J, Vallari AS, Justman J (2013). Use of a multifaceted approach to analyze HIV incidence in a cohort study of women in the United States: HIV prevention trials network 064 study. J Infect Dis.

[6] Brown T, Grassly NC, Garnett G, Stanecki K (2006). Improving projections at the country level: the UNAIDS estimation and projection package. 2005. Sex Transm Infect.

[7] Kassanjee R, Pilcher CD, Keating SM, Facente SN, McKinney E, Price MA, Martin JN, Little S, Hecht FM, Kallas EG (2014). CEPHIA: independent assessment of candidate HIV incidence assays on specimens in the CEPHIA repository. AIDS.

[8] Laeyendecker O, Brookmeyer R, Oliver AE, Mullis CE, Eaton KP, Mueller AC, Jacobson LP, Margolick JB, Brown J, Rinaldo CR (2012). Factors associated with incorrect identification of recent HIV infection using the BED capture immunoassay. Aids Res Hum Retrov.

[9] Marinda ET, Hargrove J, Preiser W, Slabbert H, van Zyl G, Levin J, Moulton LH, Welte A, Humphrey J (2010). Significantly diminished long-term specificity of the BED capture enzyme immunoassay among patients with HIV-1 with very low CD4 counts and those on antiretroviral therapy. J Acq Imm Def.

[10] Longosz AF, Mehta SH, Kirk GD, Margolick JB, Brown J, Quinn TC, Eshleman SH, Laeyendecker O (2014). Incorrect identification of recent HIV infection in adults in the United States using a limiting-antigen avidity assay. AIDS.

[11] Wendel SK, Mullis CE, Eshleman SH, Blankson JN, Moore RD, Keruly JC, Brookmeyer R, Quinn TC, Laeyendecker O. Effect of natural and ARV-induced viral suppression and viral breakthrough on anti-HIV antibody proportion and avidity in patients with HIV-1 subtype B infection. PLoS One. 2013;8(2) 10.1371/journal.pone.0055525. 10.1371/journal.pone.0055525 PMC3577851 23437058

[12] Huynh D, Laeyendecker O, Brookmeyer R (2014). A serial risk score approach to disease classification that accounts for accuracy and cost. Biometrics.

[13] Van Beckhoven D, Buve A, Ruelle J, Seyler L, Sasse A (2012). A national cohort of HIV-infected patients in Belgium: design and main characteristics. Acta Clin Belg.

[14] de Oliveira T, Deforche K, Cassol S, Salminen M, Paraskevis D, Seebregts C, Snoeck J, van Rensburg EJ, Wensing AM, van de Vijver DA (2005). An automated genotyping system for analysis of HIV-1 and other microbial sequences. Bioinformatics.

[15] Struck D, Lawyer G, Ternes AM, Schmit JC, Bercoff DP (2014). COMET: adaptive context-based modeling for ultrafast HIV-1 subtype identification. Nucleic Acids Res.

[16] Brookmeyer R, Quinn TC (1995). Estimation of current human-immunodeficiency-virus incidence rates from a cross-sectional survey using early diagnostic-tests. Am J Epidemiol.

[17] Fiebig EW, Wright DJ, Rawal BD, Garrett PE, Schumacher RT, Peddada L, Heldebrant C, Smith R, Conrad A, Kleinman SH (2003). Dynamics of HIV viremia and antibody seroconversion in plasma donors: implications for diagnosis and staging of primary HIV infection. AIDS.

[18] Taylor D, Durigon M, Davis H, Archibald C, Konrad B, Coombs D, Gilbert M, Cook D, Krajden M, Wong T (2015). Probability of a false-negative HIV antibody test result during the window period: a tool for pre- and post-test counselling. Int J STD AIDS.

[19] Hauser A, Santos-Hoevener C, Meixenberger K, Zimmermann R, Somogyi S, Fiedler S, Hofmann A, Bartmeyer B, Jansen K, Hamouda O, et al. Improved testing of recent HIV-1 infections with the BioRad avidity assay compared to the limiting antigen avidity assay and BED capture enzyme immunoassay: evaluation using reference sample panels from the German Seroconverter cohort. PLoS One. 2014;9(6) 10.1371/journal.pone.0098038. 10.1371/journal.pone.0098038 PMC4043688 24892795

[20] Parekh BS, Hanson DL, Hargrove J, Branson B, Green T, Dobbs T, Constantine N, Overbaugh J, McDougal JS (2011). Determination of mean Recency period for estimation of HIV type 1 incidence with the BED-capture EIA in persons infected with diverse subtypes. Aids Res Hum Retrov.

[21] Hanson DL, Song R, Masciotra S, Hernandez A, Dobbs TL, Parekh BS, Owen SM, Green TA (2016). Mean Recency period for estimation of HIV-1 incidence with the BED-capture EIA and bio-Rad avidity in persons diagnosed in the United States with subtype B. PLoS One.

[22] Busch MP, Pilcher CD, Mastro TD, Kaldor J, Vercauteren G, Rodriguez W, Rousseau C, Rehle TM, Welte A, Averill MD (2010). Incidence WWGH: beyond detuning: 10 years of progress and new challenges in the development and application of assays for HIV incidence estimation. AIDS.

[23] Hallett TB (2011). Estimating the HIV incidence rate: recent and future developments. Curr Opin HIV AIDS.

[24] Welte A, McWalter TA, Laeyendecker O, Hallett TB (2010). Using tests for recent infection to estimate incidence: problems and prospects for HIV. Eur Secur.

[25] Kassanjee R, Pilcher CD, Busch MP, Murphy G, Facente SN, Keating SM, McKinney E, Marson K, Price MA, Martin JN (2016). Viral load criteria and threshold optimization to improve HIV incidence assay characteristics. AIDS.

[26] Konikoff J, Brookmeyer R, Longosz AF, Cousins MM, Celum C, Buchbinder SP, Seage GR, Kirk GD, Moore RD, Mehta SH, et al. Performance of a limiting-antigen avidity enzyme immunoassay for cross-sectional estimation of HIV incidence in the United States. PLoS One. 2013;8(12) 10.1371/journal.pone.0082772. 10.1371/journal.pone.0082772 PMC3873916 24386116

[27] Hladik W, Olara D, Mermin J, Moore D, Were W, Alexander L, Downing R (2012). Effect of CD4(+) T cell count and antiretroviral treatment on two serological HIV incidence assays. Aids Res Hum Retrov.

[28] Henrard DR, Daar E, Farzadegan H, Clark SJ, Phillips J, Shaw GM, Busch MP (1995). Virological and immunological characterization of symptomatic and asymptomatic primary Hiv-1 infection. J Acquir Immune Defic Syndr Human Retrovirol.

[29] Sasse A, Florence E, Pharris A, De Wit S, Lacor P, Van Beckhoven D, Deblonde J, Delforge ML, Fransen K, Goffard JC (2016). Late presentation to HIV testing is overestimated when based on the consensus definition. HIV Med.

[30] Sudha T, Lakshmi V, Teja VD (2006). Western blot profile in HIV infection. Indian J Dermatol Venereol Leprol.

[31] Yu L, Laeyendecker O, Wendel SK, Liang FX, Liu W, Wang XY, Wang L, Pang XW, Fang ZL (2015). Short communication: low false recent rate of limiting-antigen avidity assay among long-term infected subjects from Guangxi, China. Aids Res Hum Retrov.

[32] Dauwe K, Mortier V, Schauvliege M, Van den Heuvel A, Fransen K, Servais JY, Bercoff DP, Seguin-Devaux C, Verhofstede C (2015). Characteristics and spread to the native population of HIV-1 non-B subtypes in two European countries with high migration rate. BMC Infect Dis.

[33] Mullis CE, Munshaw S, Grabowski MK, Eshleman SH, Serwadda D, Brookmeyer R, Nalugoda F, Kigozi G, Kagaayi J, Tobian AAR (2013). Differential specificity of HIV incidence assays in HIV subtypes a and D-infected individuals from Rakai, Uganda. Aids Res Hum Retrov.

[34] Longosz AF, Serwadda D, Nalugoda F, Kigozi G, Franco V, Gray RH, Quinn TC, Eshleman SH, Laeyendecker O (2014). Impact of HIV subtype on performance of the limiting antigen-avidity enzyme immunoassay, the bio-Rad avidity assay, and the BED capture immunoassay in Rakai, Uganda. Aids Res Hum Retrov.

[35] Cousins MM, Konikoff J, Sabin D, Khaki L, Longosz AF, Laeyendecker O, Celum C, Buchbinder SP, Seage GR, Kirk GD, et al. A comparison of two measures of HIV diversity in multi-assay algorithms for HIV incidence estimation. PLoS One. 2014;9(6) 10.1371/journal.pone.0101043. 10.1371/journal.pone.0101043 PMC4072769 24968135

[36] Braunstein SL, Nash D, Kim AA, Ford K, Mwambarangwe L, Ingabire CM, Vyankandondera J, van de Wijgert JH (2011). Dual testing algorithm of BED-CEIA and AxSYM avidity index assays performs best in identifying recent HIV infection in a sample of Rwandan sex workers. PLoS One.

